# Treating to the target of remission in early rheumatoid arthritis is cost-effective: results of the DREAM registry

**DOI:** 10.1186/1471-2474-14-350

**Published:** 2013-12-13

**Authors:** Marloes Vermeer, Wietske Kievit, Hillechiena H Kuper, Louise MA Braakman-Jansen, Hein J Bernelot Moens, Theo R Zijlstra, Alfons A den Broeder, Piet LCM van Riel, Jaap Fransen, Mart AFJ van de Laar

**Affiliations:** 1 Arthritis Center Twente, Department of Psychology, Health and Technology, University of Twente, Enschede, The Netherlands; 2 Department of Rheumatology and Clinical Immunology, Medisch Spectrum Twente, Enschede, The Netherlands; 3 Department of Rheumatology, Radboud University Nijmegen Medical Centre, Nijmegen, The Netherlands; 4 Department of Rheumatology, Ziekenhuisgroep Twente, Hengelo, The Netherlands; 5 Department of Rheumatology, Isala Klinieken, Zwolle, The Netherlands; 6 Department of Rheumatology, Maartenskliniek, Nijmegen, The Netherlands

## Abstract

**Background:**

Where health economic studies are frequently performed using modelling, with input from randomized controlled trials and best guesses, we used real-life data to analyse the cost-effectiveness and cost-utility of a treatment strategy aiming to the target of remission compared to usual care in early rheumatoid arthritis (RA).

**Methods:**

We used real-life data from comparable cohorts in the Dutch Rheumatoid Arthritis Monitoring (DREAM) registry: the DREAM remission induction cohort (treat-to-target, T2T) and the Nijmegen early RA inception cohort (usual care, UC). Both cohorts were followed prospectively using the DREAM registry methodology. All patients fulfilled the American College of Rheumatology criteria for RA and were included in the cohort at the time of diagnosis. The T2T cohort was treated according to a protocolised strategy aiming at remission (Disease Activity Score in 28 joints (DAS28) < 2.6). The UC cohort was treated without DAS28-guided treatment decisions. EuroQol-5D utility scores were estimated from the Health Assessment Questionnaire. A health care perspective was adopted and direct medical costs were collected. The incremental cost effectiveness ratio (ICER) per patient in remission and incremental cost utility ratio (ICUR) per quality-adjusted life year (QALY) gained were calculated over two and three years of follow-up.

**Results:**

Two year data were available for 261 T2T patients and 213 UC patients; an extended follow-up of three years was available for 127 and 180 patients, respectively. T2T produced higher remission percentages and a larger gain in QALYs than UC. The ICER was € 3,591 per patient in remission after two years and T2T was dominant after three years. The ICUR was € 19,410 per QALY after two years and T2T was dominant after three years.

**Conclusions:**

We can conclude that treating to the target of remission in early RA is cost-effective compared with UC. The data suggest that in the third year, T2T becomes cost-saving.

## Background

Treating to the target of remission has become the new paradigm for the treatment of patients with rheumatoid arthritis (RA) [1]. The key elements of treat-to-target (T2T) are: monitoring disease activity, subsequently adjusting medication in accordance to a fixed protocol, and aiming at a predefined target. In clinical trials it has been demonstrated that a T2T approach is more effective in lowering disease activity and, ultimately, reaching remission than usual care [2-7].

Taking into account that treating RA comes with potential high costs, it is mandatory to study the balance between costs and effects and ultimately gained quality of life. Health economic studies addressing this question frequently use modelling as methodology to evaluate this balance. These studies use data from pivotal trials and best guesses by opinion leaders to feed the model. For prediction before or early after introducing innovations to the market, modelling is a realistic approach. However, clinical trial data, clinical experience and mathematical models have their restrictions. Therefore, real-life data are needed to study the economic impact of innovations in health care compared with usual care.

In the Dutch Rheumatoid Arthritis Monitoring (DREAM) registry, 11 centres prospectively acquire standardized data on their RA patients. In the DREAM registry, centres participate in different levels and cohorts. One of the DREAM cohorts is the DREAM remission induction cohort. With this cohort we have demonstrated that a T2T strategy aiming for remission (Disease Activity Score in 28 joints (DAS28) < 2.6 [8]) is very effective in daily clinical practice, with percentages of DAS28 remission ranging from 47% after six months to 58% after twelve months [9]. In this early RA cohort, remission was achieved rapidly with a median time to first remission of 25 weeks. Moreover, this T2T strategy resulted in beneficial clinical outcomes after one year compared to usual care treatment [7].

Early and effective suppression of disease activity is expected to reduce pain, prevent progression of joint damage and disability [10,11], and increase the patient’s quality of life [12,13]. The concept of T2T assumes that intensive efforts and costs are made in the beginning of the disease to gain health and cost savings later. However, the question is whether indeed the health benefits outweigh the extra costs associated with performing a T2T approach.

The objective of this health economic study is to evaluate the cost-effectiveness and cost-utility, from a health care perspective, of a T2T strategy aiming at remission compared to usual care for the treatment of early RA patients in real-life daily clinical practice over a period of up to three years.

## Methods

### Study design

The data in this study are prospectively acquired in participating centres of the DREAM registry. Post-hoc we analysed the data of two cohorts. All DREAM centres are stationed in the eastern part of The Netherlands and have the same health care and reimbursement system. The T2T cohort consisted of patients from the DREAM remission induction cohort and the usual care (UC) cohort consisted of patients from the Nijmegen early RA inception cohort [14]. This study can be defined as a quasi-experiment because unselected patients were included in both cohorts with ‘living area’ as main determinant for being included in either one of the cohorts. In both cohorts, all clinical data on patient characteristics, medication, clinical and laboratory measures were assessed in a standardized way and stored prospectively in electronic databases. Currently, in both cohorts, new patients are still being included and follow-up continues. The local medical ethics committees have approved the prospective data acquisition of both inception cohorts (Medisch Spectrum Twente Hospital for the T2T cohort and CMO region Arnhem-Nijmegen for the usual care cohort). Each patient gave informed consent before the inclusion in the cohorts.

### DREAM registry - treat-to-target

Since January 2006, patients were enrolled in the DREAM remission induction cohort [9]. A T2T strategy including standardised and protocolised treatment adjustments aiming at remission (DAS28 < 2.6) was applied. Patients visited the clinic at weeks 0, 8, 12, 20, 24, 36 and 52, and every three months thereafter. Therapy consisted of initial methotrexate monotherapy (MTX), followed by the addition of sulfasalazine (SSZ), and thereafter in the case of persistent disease activity, sulfasalazine was replaced with anti-tumour necrosis factor (TNF) α agents. If the target of DAS28 < 2.6 was met, medication was not changed. In case of sustained remission (≥ six months), medication was gradually reduced and eventually discontinued. Nonsteroidal anti-inflammatory drugs (NSAIDs), prednisolone at a dosage of ≤ 10 mg/day, and intra-articular corticosteroid injections were allowed at the discretion of the attending rheumatologist. Data collection, including assessment of the DAS28, was performed by trained rheumatology nurses.

### DREAM registry - usual care

Follow-up and data acquisition for the usual care cohort were similar to the T2T cohort in the DREAM registry. Every three months, the DAS28 was assessed by trained rheumatology nurses. In contrast to a T2T approach, the DAS28 values were not generally provided to the treating rheumatologist and pharmacological treatment was not protocolised but at the discretion of the rheumatologist. In general, patients were treated with step-up or sequential monotherapy with conventional disease-modifying anti-rheumatic drugs (DMARDs) and/or biologic, notably anti-TNF. Prednisolone (oral or injections) and NSAIDs could also be used. The most commonly applied strategy was starting with MTX monotherapy, subsequently switching to SSZ or adding SSZ in case of MTX failure, and adding an anti-TNF agent after two or more DMARDs had failed.

### Selection of patients

For the current study, we selected patients from both cohorts who fulfilled the following inclusion criterion: RA according to the American College of Rheumatology 1987 classification criteria for RA [15], age ≥ 18 years, symptom duration of less than one year, and no previous treatment with DMARDs. Patients diagnosed between January 2000 and February 2009 with a minimal follow-up of two years were selected. The DREAM remission induction cohort started in 2006. Although we hoped to have sufficient data on UC in a comparable time slot, we had to include patients for the UC group diagnosed from January 2000 onwards in order to obtain a sufficient number of patients. We chose 2000 as a minimum year of inclusion because since then biologic agents, especially TNF blockers, were already available and reimbursed with restriction in daily practice.

Patients from the T2T cohort visited Arthritis Center Twente at Medisch Spectrum Twente, Enschede and the departments of rheumatology from Ziekenhuisgroep Twente, Almelo/Hengelo and Isala Klinieken, Zwolle. Patients from the UC cohort visited the Radboud University Nijmegen Medical Centre or Maartenskliniek, Nijmegen.

### Measurements

The effectiveness of treatment was evaluated using the DAS28 (calculated using the erythrocyte sedimentation rate (ESR)). A DAS28 < 2.6 was defined as remission [16].

Utilities were estimated to evaluate the effect of treatment on health-related quality of life. Utility is the valuation of a health state on a scale of 0 (equivalent to death) to 1 (equivalent to full health) and is used to derive quality-adjusted life years (QALYs) [17]. Because preference based measures were not prospectively assessed, EuroQol-5D (EQ-5D) values [18] were estimated from the Health Assessment Questionnaire (HAQ) scores [19,20] by using model 5 of the mapping method by Bansback et al. [21]. This model was reported to be the most successful of the five mapping methods, by having the lowest mean square error and the best predictive value [21,22]. Concordantly, the QALYs were computed according to the trapezium rule.

### Cost analysis

The cost analysis exists of two main parts. First, on patient level, volumes of care related to the T2T strategy or usual care were determined. Volumes of hospital related care, i.e. consultations with the rheumatologist and the rheumatology nurse, telephonic consultations (rheumatologist), and hospital admissions related to RA, were retrieved from the hospital information system. Medication use (exact dose of medication and administration period) was prospectively registered in the electronic case report forms.

The second part of the cost analysis consisted of determining the cost prices for each volume of consumption. Volumes of care were multiplied by the cost prices for each volume of care to calculate costs. The standard cost prices from the Dutch Guideline for Cost Analyses were used for hospital related care (see Appendix) [23]. The price based on personnel, material and overhead of day care hospital admissions required for treatment with infliximab or rituximab was estimated at a mean of € 122 per day (on top of the medication costs). Cost prices for medication were retrieved from the Dutch national tariff list provided by the Dutch Board of Health Insurances [23].

The base year was 2011 for all prices. Prices retrieved from other years were converted to 2011 euros using the general Dutch price index rate [24].

### Statistical analysis

Data of two year follow-up were analysed as well as an extended follow-up of three years in patients who had sufficient follow-up. Our expectation was that on the long-term, costs associated with performing T2T will decrease. In our previous study, the necessary sample size to detect a difference in remission of 20% between both groups was estimated to be at least 2×125 = 250 patients [7]. This sample size estimation was satisfied in the two and three years data analyses in the present study.

The incremental cost-effectiveness ratio (ICER) was calculated by dividing the difference in costs by the difference in effectiveness (based on the number of patients in remission) derived from the two groups. The ICER is expressed as costs per one more patient in remission. The incremental cost-utility ratio (ICUR) was calculated by dividing the difference in costs by the difference in the QALYs produced by the two groups. The ICUR is expressed as costs per QALY gained. Uncertainty in both ratios was determined non-parametrically using bootstrap techniques. Results of the 1,000 bootstrapped replications are presented in cost-effectiveness planes that graphically present the uncertainty around the ratio of the two and three years data. In a sub-analysis, the ratios were calculated for patients that were included in the cohorts from 2006. However, statistical power was expected to be low due to the low number of UC patients.

Missing values were imputed with single imputation using a regression method including a random component for the ESR, patient’s assessment of general health, and HAQ question 23 (take a tub bath) or linear interpolation using the trapezoid method for the DAS28 and EQ-5D scores, conditional on the data being missing at random.

The level of significance was set at a p value < 0.05. Statistical analyses were performed using the statistical software package SPSS 18.0 (SPSS Inc., Chicago, IL, USA). The bootstrap was performed in Excel.

## Results

### Baseline characteristics

Two year follow-up data were available for 261 patients of the T2T cohort and for 213 patients of the UC cohort. An extended three years follow-up was available for a smaller proportion of patients due to insufficient follow-up; i.e. in 127 of the 261 (48.7%) T2T patients and 180 of the 213 (84.5%) UC patients. Baseline characteristics were comparable between patients with and without sufficient follow-up.

Table 1 presents the demographic and clinical characteristics of both groups at baseline. The groups were comparable at baseline regarding age, gender, rheumatoid factor (RF) positivity, number of tender joints (28 assessed) and ESR. Statistically significant but small differences were found for the mean DAS28, number of swollen joints (28 assessed), C-reactive protein, patient’s assessment of general health and pain, and HAQ score, which were higher in the T2T group.

**Table 1:** Baseline characteristics of the patients of the treat-to-target (T2T) and usual care (UC) groups

	**T2T (n=261)**	**UC (n=213)**	**UC from 2006 (n=69)**
Age, mean ± SD years	57.9 ± 13.8	56.6 ±13.4	53.9 ±13.0†
Female sex, n (%)	161 (61.7)	132 (62.0)	43 (62.3)
RF positive, n (%)	178 (68.2)	147/211 (69.7)	48/69 (69.6)
DAS28, mean ± SD	5.0 ± 1.1	4.8 ± 1.2†	4.8 ± 1.3
No. of swollen joints (28 assessed), median (IQR)	8 (5–12)	9 (6–13)†	8 (5–12)
No. of tender joints (28 assessed), median (IQR)	5 (2–9)	4 (2–9)	4 (1–9)
ESR, median (IQR) mm/hour	28.0 (15.5-42.0)	26.0 (12.0-39.0)	29.0 (17.0-43.5)
CRP, median (IQR) mg/litre	14.0 (5.0-34.5)	6.7 (0.0-27.8)†	10.0 (0.0-34.3)
VAS general health, mean ± SD (0–100)	52.9 ± 22.6	45.7 ± 23.0†	42.9 ± 24.4†
VAS pain, mean ± SD (0–100)	51.2 ± 21.9	44.9 ± 23.2†	46.5 ± 23.9
HAQ score, median (IQR)	1.1 (0.6-1.5) (n=244)	0.9 (0.5-1.4) (n=151)†	0.9 (0.4-1.4) (n=52)†

CRP, C-reactive protein; DAS28, Disease Activity Score in 28 joints; ESR, erythrocyte sedimentation rate; HAQ, Health Assessment Questionnaire; IQR, interquartile range; RF, rheumatoid factor; SD, standard deviation; VAS, visual analog scale.

† P < 0.05 for differences between groups (T2T versus UC).

MTX monotherapy was the initial treatment in the T2T group by protocol. In the UC group, patients started with SSZ monotherapy (45.5%, 97/213), MTX monotherapy (43.7%, 93/213) or occasionally another DMARD (6.6%, 14/213) or no medication (4.2%, 9/213).

### Health outcomes

Table 2 presents the health outcome results after two and three years of follow-up. After two years, 64.4% (168/261) of the T2T group was in remission versus 34.7% (74/213) of the UC group (p < 0.001). Over the first two years of treatment, the median (interquartile range, IQR) of QALYs was higher in the T2T group than in the UC group (1.45 (1.24-1.55) versus 1.39 (1.18-1.53), respectively, p = 0.04).

**Table 2:** Health outcomes in the treat-to-target (T2T) and usual care (UC) groups after two and three years of follow-up

	**Two years**			**Three years**		
	**T2T (n=261)**	**UC (n=213)**	**UC from 2006 (n=69)**	**T2T (n=127)**	**UC (n=180)**	**UC from 2006 (n=45)**
DAS28, mean ± SD	2.4 ± 1.0	3.1 ± 1.3†	2.8 ± 1.1†	2.5 ± 1.0	3.1 ± 1.3†	2.7 ± 1.1
DAS28 level, n (%)						
Remission (DAS28 < 2.6)	168 (64.4)	74 (34.7)†	33 (47.8)†	76 (59.8)	63 (35.0)†	25 (55.6)
Low (2.6 ≤ DAS28 ≤ 3.2)	48 (18.4)	44 (20.7)	14 (20.3)	28 (22.0)	35 (19.4)	5 (11.1)
Moderate (3.2 < DAS28 ≤ 5.1)	37 (14.2)	76 (35.7)†	19 (27.5)†	21 (16.5)	72 (40.0)†	14 (31.1)†
High (DAS28 > 5.1)	8 (3.1)	19 (8.9)†	3 (4.3)	2 (1.6)	10 (5.6)	1 (2.2)
QALYs, median (IQR)	1.45 (1.24-1.55) (n=221)	1.39 (1.18-1.53) (n=143)†	1.44 (1.21-1.55) (n=47)	2.19 (1.81-2.34) (n=101)	2.04 (1.64-2.27) (n=106)†	1.95 (1.50-2.34) (n=18)

Quality-adjusted life years (QALYs) were derived from the EuroQol-5D utility scores which were estimated from the Health Assessment Questionnaire (HAQ). QALYs could not be evaluated in all patients due to missing data in (items of) the HAQ.

DAS28, Disease Activity Score in 28 joints; SD, standard deviation.

† P < 0.05 for differences between groups (T2T versus UC).

After three years, the remission percentages were 59.8% (76/127) with T2T versus 35.0% (63/180) with UC (p < 0.001). The median (IQR) of QALYs over the first three years was higher in the T2T group than in the UC group (2.19 (1.81-2.34) versus 2.04 (1.64-2.27), respectively, p = 0.05).

### Care consumption and costs

Table 3 presents the amount of care consumption and mean costs per patient during two and three years of follow-up. Over both periods, the numbers of consultations with the rheumatologist were comparable, whereas the numbers of consultations with the rheumatology nurse and telephonic consultations were higher in the T2T group. In usual care, more hospital admissions were observed than with T2T.

**Table 3:** Mean volumes of care and total direct costs in euros per patient per period in the treat-to-target (T2T) and usual care (UC) groups after two and three years of follow-up

	**0-2 year**							
	**T2T (n=261)**		**UC (n=213)**			**UC from 2006 (n=69)**		
	**Volume**	**Costs**	**Volume**	**Costs**	**Difference in costs**	**Volume**	**Costs**	**Difference in costs**
Consultations rheumatologist	10.4 ± 3.0	696 ± 199	10.3 ± 2.9	689 ± 195	7	10.2 ± 3.6	683 ± 239	13
Consultations nurse	8.8 ± 3.0	588 ± 200	7.8 ± 1.9†	522 ± 127†	66	6.6 ± 2.0†	438 ± 134†	150
Telephonic consultations	1.3 ± 1.8	34 ± 46	0.6 ± 1.4†	16 ± 36†	18	1.1 ± 1.9	29 ± 48	5
Hospital admissions	0.4 ± 2.7	178 ± 1,208	1.1 ± 4.2†	521 ± 1,901†	-343	0.3 ± 1.6	158 ± 714	20
Medication								
DMARDs/other		174 ± 165		249 ± 340	-75		166 ± 119	8
Anti-TNF		3,121 ± 7,162		1,730 ± 4,905†	1,391		1,877 ± 5,086	1244
Total		4,791 ± 7,436		3,727 ± 5,773	1,064 (1,026 to 1,121)‡		3,351 ± 5,179†	1440 (1,387 to 1479)‡
	**0-3 year**							
	**T2T (n=127)**		**UC (n=180)**			**UC from 2006 (n=45)**		
	**Volume**	**Costs**	**Volume**	**Costs**	**Difference in costs**	**Volume**	**Costs**	**Difference in costs**
Consultations rheumatologist	13.7 ± 3.3	917 ± 220	13.6 ± 3.3	909 ± 224	8	12.5 ± 3.8	838 ± 253†	79
Consultations nurse	12.1 ± 3.6	809 ± 244	8.9 ± 1.7†	596 ± 117†	213	8.4 ± 2.2†	565 ± 145†	244
Telephonic consultations	1.9 ± 2.1	49 ± 54	0.8 ± 1.7†	21 ± 45†	28	1.7 ± 2.5	43 ± 64	6
Hospital admissions	0.5 ± 3.2	215 ± 1,441	1.6 ± 5.0†	748 ± 2,263†	-533	0.8 ± 2.7	364 ± 1,217	-149
Medication								
DMARDs/other		260 ± 335		423 ± 612†	-163		259 ± 174	1
Anti-TNF		4,160 ± 10,685		4,175 ± 10,070	-15		5,488 ± 11,528	-1,328
Total		6,410 ± 10,845		6,872 ± 11,033	-462 (-513 to -350)‡		7,558 ± 11,649	-1,148 (-1241 to -1075)‡

Volumes are the mean ± standard deviation (SD).

Anti-TNF, anti-tumour necrosis factor; CI, confidence interval; DMARDs, disease-modifying antirheumatic drugs.

† P < 0.05 for differences between groups (T2T versus UC). ‡ 95% confidence interval bootstrapped.

Over the first two years, the mean (standard deviation, SD) total direct costs per patient were € 4,791 (7,436) in the T2T group and € 3,727 (5,773) in the usual care group (Table 3). The observed difference in costs between groups was mainly generated by the costs of anti-TNF therapy and hospitalization. During the first two years of treatment, 21.5% (56/261) of the T2T group received anti-TNF therapy versus 15.0% (32/213) of the UC group.

Over the first three years, the mean (SD) total direct costs per patient were € 6,410 (10,845) in the T2T group and € 6,872 (11,033) in the UC group (Table 3). The observed difference in costs between groups was mainly generated by hospitalization.

Overall, the mean (SD) time until the first anti-TNF agent was started was shorter in the T2T group compared to the UC group (mean (SD) of 58 (29) weeks versus 80 (39) weeks, respectively, p = 0.002).

### Cost-effectiveness

**Figure 1:**
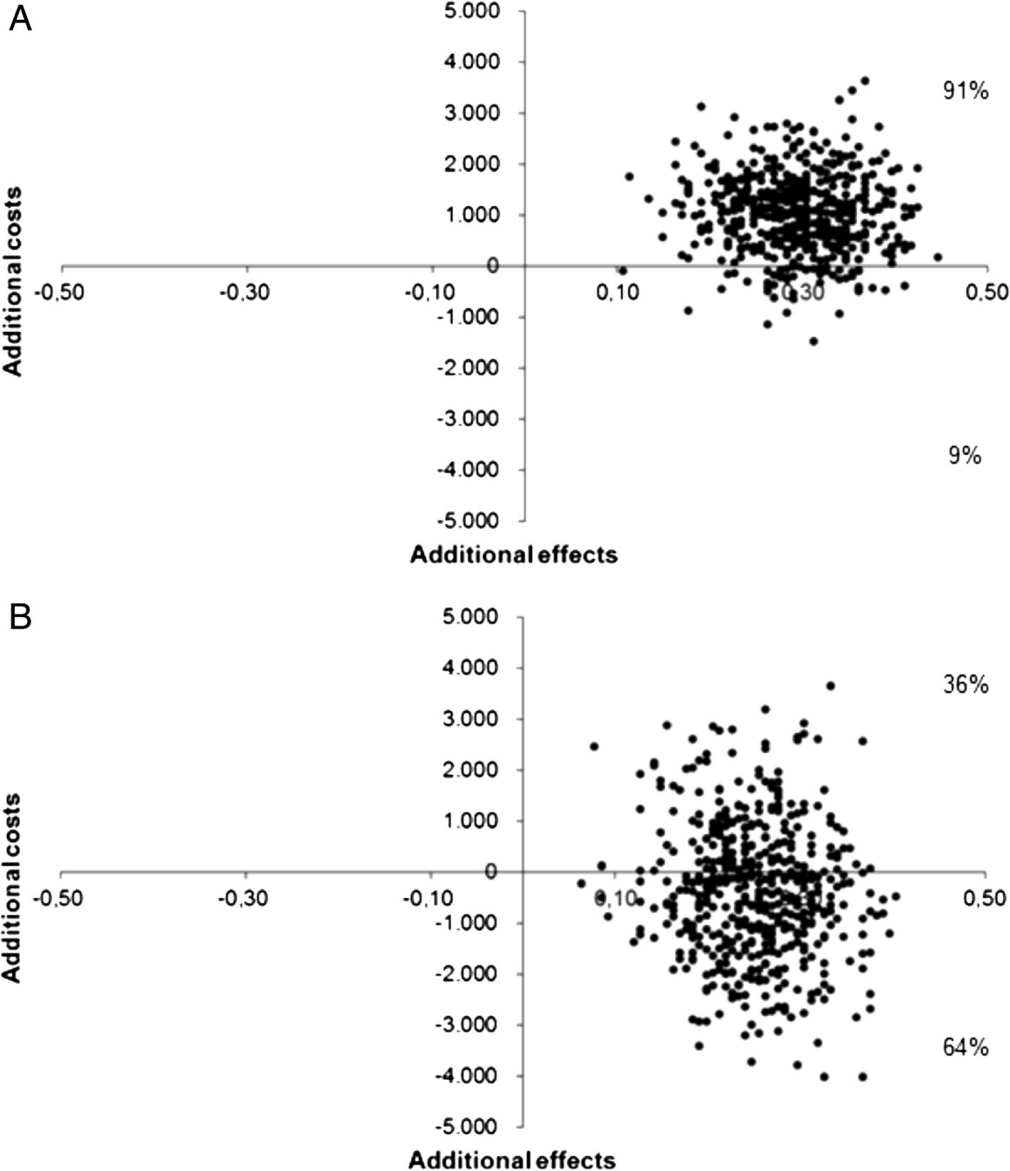
Cost-effectiveness planes of 1000 bootstrap replicates of the incremental cost and effectiveness (based on the number of patients in remission, defined as a Disease Activity Score in 28 joints < 2.6) of the treat-to-target strategy versus usual care in early rheumatoid arthritis after A) two years and B) three years of follow-up.

After two years of follow-up, the ICER was € 3,591 per patient in remission and after three years of follow-up the T2T strategy was dominant. Figure 1 presents the cost-effectiveness planes, showing the relation between the difference in effect (x-axis) and the difference in costs (y-axis) between T2T and UC. Figure 1A presents the two years data and shows that 91% of the bootstrapped ratios were situated in the upper-right quadrant, which signifies a gain in effectiveness against higher costs. Figure 1B shows that after three years, 64% of the bootstrapped ratios were situated in the lower-right quadrant, which signifies lower costs and higher effectiveness.

### Cost-utility

**Figure 2:**
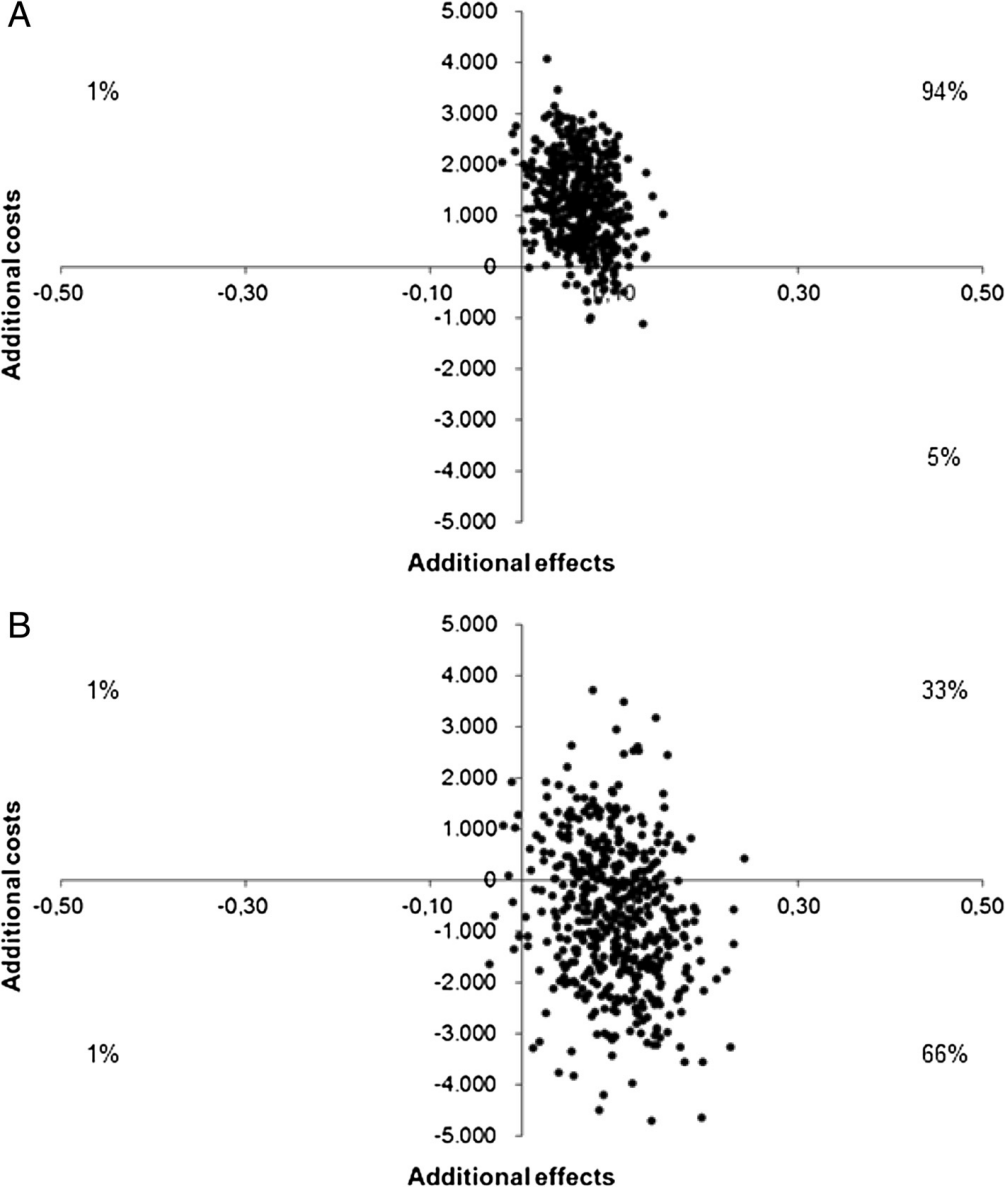
Cost-utility planes of 1000 bootstrap replicates of the incremental cost and quality-adjusted life years gained by the treat-to-target strategy versus usual care in early rheumatoid arthritis after A) two and B) three years of follow-up.

Over a period of two years, the ICUR was € 19,410 per QALY and after three years of treatment the T2T strategy was dominant. Figure 2 presents the cost-utility planes, showing the relation between the difference in QALYs (x-axis) and the difference in costs (y-axis) between T2T and UC. Figure 2A shows that after 94% of the two years’ bootstrapped ratios were situated in the upper-right quadrant. Figure 2B shows that 66% of the three years’ bootstrapped ratios were situated in the lower-right quadrant, which signifies lower costs and higher effectiveness.

### Sub-analysis

A sub-analysis was performed on patients that were included in the cohorts from 2006. Table 1 presents the baseline characteristics of the 69 UC patients that were included in the UC cohort from 2006. The health outcomes of these patients are presented in Table 2 and the amount of care consumption and mean costs per patient during two and three years of follow-up are presented in Table 3. After two years of follow-up, the ICER was € 8,709 per patient in remission. After three years T2T was dominant (this analysis included 45 UC patients). We were not able to perform analysis on the ICURs because of the low number of patients with data with on QALYs in the UC group.

## Discussion

Our study suggests that treating to the target of remission is the preferred strategy over usual care in early RA. After two years of treatment, T2T is cost-effective as it comes with higher costs but also with substantially higher effectiveness. In the T2T group DAS28 remission had been achieved more frequently and there was a larger gain in health-related QALYs compared with UC. The ICUR lies far below the threshold of €80,000 per QALY, which is considered to be an acceptable willingness to pay for one QALY in The Netherlands [25]. Moreover, the costs to bring one more patient in remission also seem to be acceptable. Results of an extended follow-up analysis of three year data were clearly in favor of T2T, with 64% chance of the T2T strategy coming at lower costs with higher effectiveness compared to UC. To our knowledge, this is the first health economic evaluation of comparing T2T with UC using real-life data.

The drivers of absolute costs and cost differences between T2T and UC were anti-TNF therapy and hospitalization. Our previous studies demonstrated that the majority of the T2T patients achieved remission with conventional DMARDs [7,9]. According to the treatment protocol, anti-TNF was prescribed only for a minority of patients whose disease activity remained moderate to high after insufficient effect of conventional DMARDs, thereby preventing overtreatment with anti-TNF agents with their costs and side effects. In the UC group, anti-TNF was initiated later in the disease course, and, therefore, it might be less effective and longer required in patients. Costs due to hospitalization were directly related to RA. The higher number of hospital admissions in the UC group might be explained by less efficient disease control. Data on intra-articular injections were not available. However, we do not think this can explain the difference in costs between T2T and UC, because of the low costs of injections.

The principle of T2T is to aim at achieving and sustaining remission as early as possible. Our expectation is that the extra effort and time spent in the first years of the disease, ultimately result in a reduction of the number of consultations later in the disease course and the possibility of tapering and discontinuing medication in case of sustained remission, thereby diminishing costs. Therefore, we expect that on the long-term, cost savings associated with T2T will increase. Furthermore, better and earlier disease control might lead to more work participation on the long-term, which will ultimately lower the costs of T2T for society and improve quality of life for the patients.

An important strength of this study is the quasi-experimental design containing real-life observational data regarding effectiveness, health-related quality of life and costs of T2T compared with usual care. Moreover, in the DREAM registry all consecutive patients are prospectively followed and a standardized data set is collected. This is in contrast to many health economic evaluations, which often use modeling techniques with many underlying assumptions or use clinical trial data of highly selected patients.

However, this study has some limitations also. Obviously, since our patients are unselected, randomisation between the comparing cohorts was not performed with the risk of confounding by indication present in the comparison. However, participation in either one of the cohorts was determined by living area while patients attended comparable rheumatology clinics, all participating in the DREAM registry and working within the same health care system. We assume that this has limited the possibility of confounding by indication. Furthermore, no relevant differences in baseline characteristics which are prognostic for the treatment effect were found. Second, it should be noted that UC patients were included from 2000 until present, whereas T2T patients were included from 2006 until present. Even though the same treatment options for both groups were available during observation and anti-TNF guidelines have not been changed since 2000, one can assume that UC has changed between 2000 and 2006. A sub-analysis omitting UC patients recruited prior to 2006 showed comparable ICERs, however with less statistical power, leading to the same conclusions. Therefore, this study provides the best possible comparison currently available. A third limitation is that a preference based health-related quality of life measure was not available in the UC cohort, and, therefore, we estimated utilities from the HAQ. The HAQ has been shown to be highly correlated with health state utility values, which are used to calculate QALYs [26]. Nevertheless, HAQ-derived utilities will only capture change in quality of life generated by the patient’s functional status and not by other factors. We expect that T2T patients improve at more dimensions of quality of life than only function status. Therefore, we believe that this was a conservative analysis. We acknowledge that the use of a mapping method will always be suboptimal to primary collection of utility data. Fourth, we applied a health care perspective, thereby taking into account only direct medical costs. However, the economic burden of RA goes beyond health care costs [27-29] and a societal perspective would be preferable. RA leads to substantial losses in terms of work productivity which increases with disease duration [30,31]. Unfortunately, data on work participation were not available. According to our view, we performed a conservative cost analysis and our expectations are that T2T, which decreases disease activity rapidly and early in the disease course, will have an additional positive effect on non-medical costs (e.g. work productivity, informal care, and paid housekeeping).

## Conclusions

We performed a health economic study of T2T versus UC, using observational data of the DREAM registry. We conclude that treating to the target of remission in early RA is cost-effective as compared with usual care at the discretion of the attending physician.

## Appendix

### Cost prices 2011

Consult at rheumatologist (13 minutes) € 66.90

Consult at rheumatology nurse (20 minutes) € 66.90

Telephonic consult at rheumatologist (5 minutes) € 25.73

Hospital day care related to biologics € 122.13

Hospital admission (one day) € 454.69

## Competing interests

An unrestricted educational grant was provided by Abbott, The Netherlands, which was used to support MV.

The authors declare that they have no competing interests.

## Authors’ contributions

MV contributed to the design of the study, data collection, analysis and interpretation, and drafted the manuscript. WK contributed to the design of the study, data collection, analysis and interpretation, and drafted the manuscript. HHK contributed to the design of the study, data collection and interpretation, and drafted the manuscript. LMAB-J contributed to the design of the study, data interpretation, and critically revised the manuscript. HJBM contributed to data collection and interpretation, and critically revised the manuscript. TRZ contributed to data collection and interpretation, and critically revised the manuscript. AAdB contributed to data collection and interpretation, and critically revised the manuscript. PLCMvR contributed to the design of the study, data collection and interpretation, and critically revised the manuscript. JF contributed to the design of the study, data interpretation, and critically revised the manuscript. MAFJvdL contributed to the design of the study, data collection and interpretation, and drafted the manuscript. All authors read and approved the final manuscript.

## Pre-publication history

The pre-publication history for this paper can be accessed here:

http://www.biomedcentral.com/1471-2474/14/350/prepub
